# Phosphonates of *Pectobacterium atrosepticum*: Discovery and Role in Plant–Pathogen Interactions

**DOI:** 10.3390/ijms252111516

**Published:** 2024-10-26

**Authors:** Olga Parfirova, Polina Mikshina, Olga Petrova, Andrey Smolobochkin, Alexander Pashagin, Alexander Burilov, Vladimir Gorshkov

**Affiliations:** 1 Kazan Institute of Biochemistry and Biophysics, Federal Research Center “Kazan Scientific Center of the Russian Academy of Sciences”, 420111 Kazan, Russia; parfirovaolga.i@gmail.com (O.P.); p.mikshina@gmail.com (P.M.); poe60@mail.ru (O.P.); pavmail73@gmail.com (A.P.); 2 Arbuzov Institute of Organic and Physical Chemistry, Federal Research Center “Kazan Scientific Center of the Russian Academy of Sciences”, 420088 Kazan, Russia; smolobochkinav@mail.ru (A.S.); burilov@iopc.ru (A.B.); 3 Institute of Fundamental Medicine and Biology, Kazan Federal University, 420008 Kazan, Russia

**Keywords:** plant soft rot, *Pectobacterium*, phosphonates, virulence, plant–pathogen interaction

## Abstract

Many phytopathogens’ gene products that contribute to plant–pathogen interactions remain unexplored. In one of the most harmful phytopathogenic bacterium *Pectobacterium atrosepticum* (*Pba*), phosphonate-related genes have been previously shown to be among the most upregulated following host plant colonization. However, phosphonates, compounds characterized by a carbon–phosphorus bond in their composition, have not been described in *Pectobacterium* species and other phytopathogenic bacteria, with the exception of *Pseudomonas syringae* and *Pantoea ananatis*. Our study aimed to determine whether *Pba* synthesizes extracellular phosphonates and, if so, to analyze their physiological functions. We demonstrated that *Pba* produces two types of extracellular phosphonates: 2-diethoxyphosphorylethanamine and phenylphosphonic acid. Notably, such structures have not been previously described among natural phosphonates. The production of *Pba* phosphonates was shown to be positively regulated by quorum sensing and in the presence of pectic compounds. *Pba* phosphonates were found to have a positive effect on *Pba* stress resistance and a negative effect on *Pba* virulence. The discovered *Pba* phosphonates are discussed as metabolites that enable *Pba* to control its “harmful properties”, thereby maintaining its ecological niche (the host plant) in a relatively functional state for an extended period.

## 1. Introduction

Plant diseases represent an important problem affecting agriculture and food safety worldwide. One obstacle to effective plant disease control is fragmental knowledge of plant–pathogen interactions and, in particular, of the set of “instruments” used by pathogens to colonize host plants and cause disease. Plant soft rot, caused by the members of the Soft Rot *Pectobacteriaceae* family, specifically *Pectobacterium* and *Dickeya* species, is one of the most devastating plant diseases [1]. Despite the fact that bacteria of *Pectobacterium* and *Dickeya* genera are some of the most extensively studied phytopathogens [2], it is evident that many gene products that contribute to plant–pathogen interactions and disease development remain unexplored, preventing the formation of a general picture of pathosystem development.

Plant cell wall-degrading enzymes (PCWDE) (predominantly those that digest pectic compounds) are objectively considered the main virulence factors of *Pectobacterium* and *Dickeya* species [3,4]. However, the production of these main virulence factors alone is not sufficient for effective plant colonization and disease development. Many other virulence factors are required for these bacteria to have full virulence: type three secretion system [5,6], type five secretion system [7], type six secretion system [8,9], coronafacic acid [10,11,12], siderophores [13,14,15], and exopolysaccharides [16].

In our previous study, using the comparative transcriptome profiling of *Pectobacterium atrosepticum* (*Pba*) under in vitro and planta conditions, we searched for “novel” *Pba* genes whose products might contribute to plant–*Pba* interactions [10]. In that study, genes related to the metabolism of phosphonates appeared to be one of the most upregulated genes in planta compared to in vitro conditions. Phosphonate-related *Pba* genes were also previously revealed among those that were upregulated in vitro following the addition of host plant extract [17].

Phosphonates represent a diverse group of compounds that have in common that they have a carbon–phosphorus bond (C-P) in their composition [18,19,20]. Phosphonates can be incorporated into lipids, polysaccharides, and glycoproteins (phosphonoconjugates), as well as represent “free” low-molecular-weight (LMW) compounds [18,19,20]. LMW phosphonates that are mostly described for streptomycetes possess physiological activities; the vast majority of them have antibiotic properties (fungicidal, herbicidal, and bactericidal) [21]. In plant pathogenic bacteria, LMW phosphonates have been described only in *Pseudomonas syringae* (fosfomycin similar to one produced by streptomycetes) [22,23] and *Pantoea ananatis* (pantaphos) [24], and only *P. ananatis* phosphonates have biological functions established. Pantaphos (2-(hydroxy[phosphono]methyl)maleate) has been shown to possess phytotoxic properties and to be required for the virulence of *P. ananatis* [25].

Phosphonates have not been described in *Pectobacterium* species, and it remained unknown whether these bacteria were indeed able to produce these compounds. Some *Pectobacterium* species, including *Pba*, have a “marker” gene encoding phosphoenolpyruvate mutase Fom1 (ECA0487) that catalyzes the formation of the C-P bond, yielding phosphonopyruvate, as well as a gene encoding phosphoenolpyruvate decarboxylase Fom2 (ECA0488), which provides the further common step of biosynthesis of most known phosphonates, yielding phosphonoaldehyde. The gene encoding transaminase, which catalyzes the conversion of phosphonoaldehyde to 2-aminoethyl phosphonate, a precursor of phosphonate-substituted lipids and glycans, has not been revealed in the *Pba* genome, whereas the gene encoding the enzyme homologous to 2-oxopropylphosphonate reductase (ECA0489), which is involved in the synthesis of LMW phosphonate fosfomycin in *Pseudomonas syringae*, is located in the *Pba* genome directly downstream of *fom1* and *fom2*. This indicates that *Pba* is more likely to produce LMW extracellular phosphonates than phosphonate-substituted lipids/polysaccharides.

Therefore, our study aimed to determine whether *Pba* produces LMW extracellular phosphonates and, if so, to analyze their role in plant–*Pba* interactions.

## 2. Results

### 2.1. Detection of Pba Phosphonates

**Figure 1:**
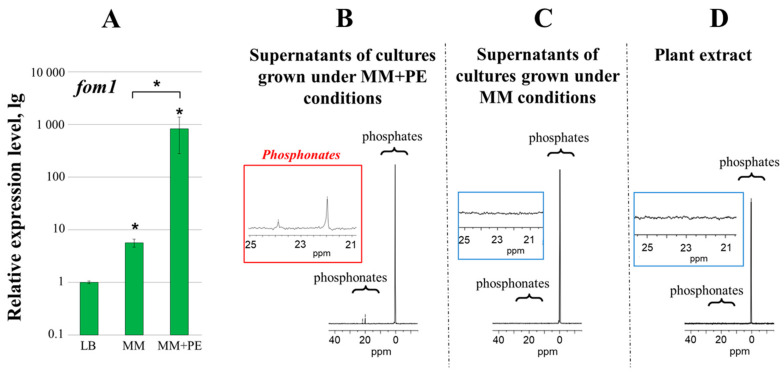
The expression levels of the *fom1* gene in *Pectobacterium atrosepticum* SCRI1043 cells (**A**) and ^31^P NMR spectra of the preparations of supernatants of *P. atrosepticum* cultures (**B**,**C**) grown under different conditions. The expression levels were determined after 24 h of growth in LB medium, minimal medium (MM), and MM supplemented with plant extract (MM + PE). The expression level of the *fom1* gene in the cells grown in LB is equated to one. Asterisks (*) show a significant difference (Mann–Whitney two-sided test, *p* < 0.05, five biological replicates) from the variant grown in LB or between the variants designated by bracket. The presence of ^31^P NMR signals at 15–25 ppm was assayed in the preparations of supernatants of cultures grown for 48 h in minimal medium (MM) and MM supplemented with plant extract (MM + PE). The ^31^P NMR spectrum of the preparation of plant extract is shown in (**D**). The detection of phosphonates was performed in at least five independent experiments.

Given that the expression of phosphonate-related genes was induced under in planta conditions [10], we analyzed the expression levels of marker gene *fom1* (encoding phosphoenolpyruvate mutase—a crucial phosphonate-biosynthetic enzyme) and detected extracellular phosphonates in vitro not only under “routine” conditions—LB medium and minimal medium (MM), but also in MM supplemented with plant extract (MM + PE). The expression level of the *fom1* gene was 800 and 150 times higher under MM + PE conditions compared to LB and MM, respectively (Figure 1A).

^31^P NMR signals in the area of δ 15–25 ppm corresponding to phosphonates were detected in the preparations of supernatants of cultures grown under MM + PE conditions but not under MM conditions (Figure 1B,C). In the preparations of plant extract, ^31^P NMR signals at 15–25 ppm have not been revealed (Figure 1D). This indicates that the phosphonates detected in the cultural supernatants were of bacterial origin but not of plant origin.

**Figure 2:**
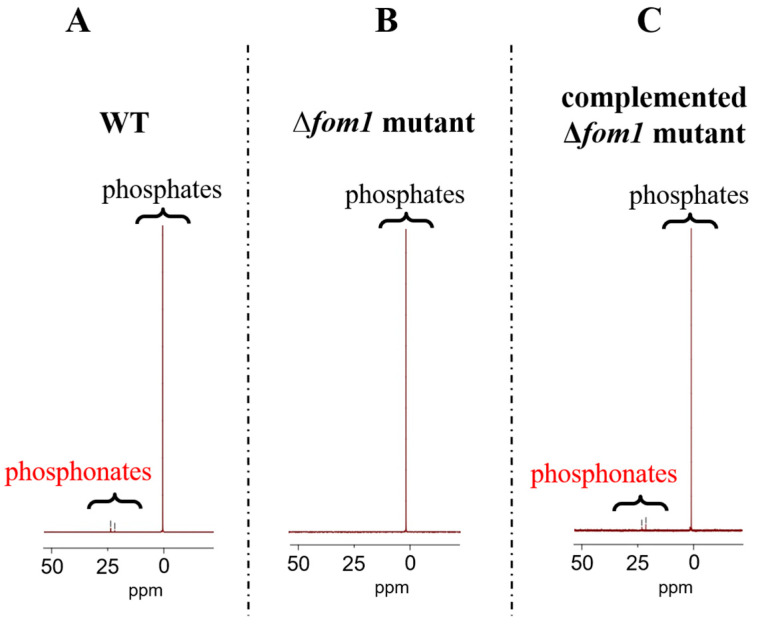
^31^P NMR spectra of the preparations of cultural supernatants of the wild-type *Pectobacterium atrosepticum* SCRI1043 (WT) (**A**), *P. atrosepticum* Δ*fom1* mutant (**B**), and complemented Δ*fom1* mutant carrying the *fom1* gene within the recombinant plasmid (**C**) grown for 48 h in minimal medium supplemented with plant extract (MM + PE). The detection of phosphonates was performed in at least five independent experiments.

To further verify the ability of *Pba* to synthesize phosphonates and to check whether the *fom1* gene indeed encodes a phosphonate-biosynthetic enzyme, the Δ*fom1* mutant and corresponding complemented Δ*fom1* mutant carrying the *fom1* gene within the recombinant plasmid were obtained. The presence of phosphonates in the cultural supernatants was assayed in wild type (WT), Δ*fom1* mutant, and the complemented Δ*fom1* mutant grown under MM + PE conditions. ^31^P NMR signals at 15–25 ppm were detected in the cultural supernatants of WT and the complemented Δ*fom1* mutant but not in the cultural supernatants of the Δ*fom1* mutant (Figure 2).

Thus, *Pba* synthesizes extracellular LMW phosphonates. The production of these compounds, along with the expression of the phosphonate-biosynthetic *fom1* gene, was induced in the presence of plant metabolites. Although the expression of the *fom1* gene was upregulated under MM conditions (without plant metabolites) compared to LB conditions, this increase (approximately 5.6-fold) was not sufficient to produce detectable levels of phosphonates.

### 2.2. Molecular Structure of Pba Phosphonates

For the determination of the molecular structure of the *Pba* phosphonates, the methanol-extractable fraction of cultural supernatant was treated with calcium acetate to remove the phosphates and analyzed by NMR spectroscopy and ESI-QTOF mass spectrometry. The analysis of the difference NMR spectra of the samples of cultural supernatants of wild-type *Pba* (producing phosphonates) and the phosphonate-deficient Δ*fom1* mutant enabled us to identify signals that corresponded to the target compounds, confirm that the target compounds were indeed phosphonates, and reveal some features of their molecular structure.

**Figure 3:**
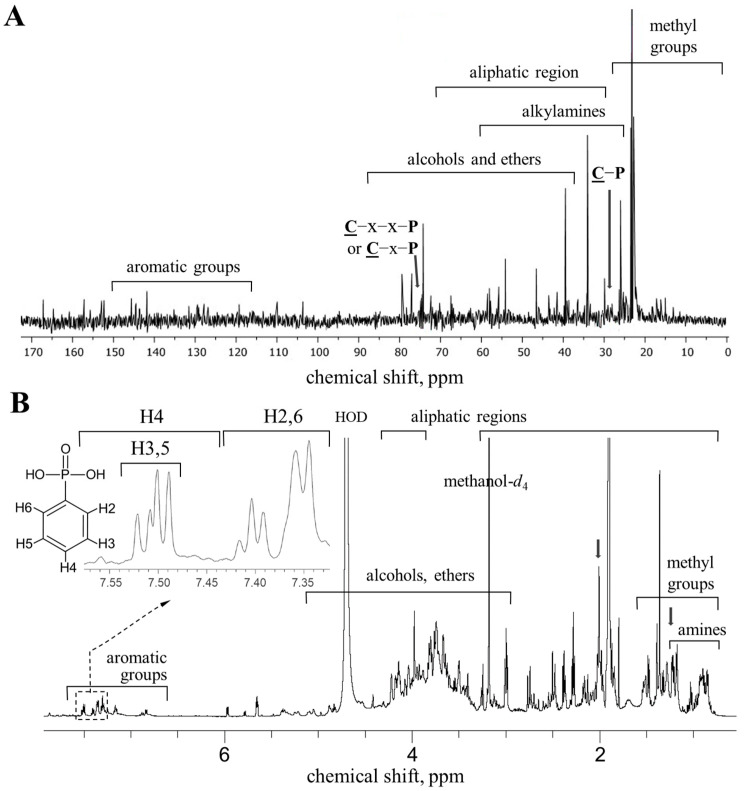
NMR spectra of the phosphonate-containing fraction of the *Pectobacterium atrosepticum* cultural supernatant. (**A**) Difference ^13^C spectrum of the cultural supernatant of wild-type *P. atrosepticum* and its phosphonate-deficient Δ*fom1* mutant. (**B**) ^1^H spectrum of the cultural supernatant of wild-type *P. atrosepticum*.

The presence of the signals in the ^13^C difference spectrum at 27–29 ppm confirmed the presence of compounds with a C-P chemical bond in the sample (Figure 3A). A doublet with a chemical shift of 74.66 ppm (J 50.9 Hz) showed the presence of a bond between a carbon atom and a phosphorus atom through 2–3 bonds. The presence of a complex multiplet in the ^1^H spectrum in the region of 1.97–2.07 ppm (Figure 3B) indicated the presence of an aliphatic fragment with nonequivalent protons. The signals at 3.0–3.5 ppm may be related to the protons of the methylene group substituted with nitrogen or oxygen atoms (Figure 3B). The possible presence of these groups was also confirmed by the signals at 25–50 ppm on the ^13^C spectrum (Figure 3A). Signals in the regions of 15–25 and 55–60 ppm (Figure 3A), as well as in the high-field region (1.2–1.5 ppm) and at 3–4 ppm (Figure 3B), suggested that the sample contained ethyl moieties. The signals in a weak field (120–150 ppm) on the ^13^C spectrum and a group of signals, including three multiplets (δ 7.38 ppm, doublets with J 8.1 Hz; δ 7.50 ppm, doublets with J 7.8 Hz; δ 7.50 ppm, doublets with J 7.7 Hz on the ^1^H spectrum, Figure 3), indicated the presence of a phenyl group in the sample.

**Figure 4:**
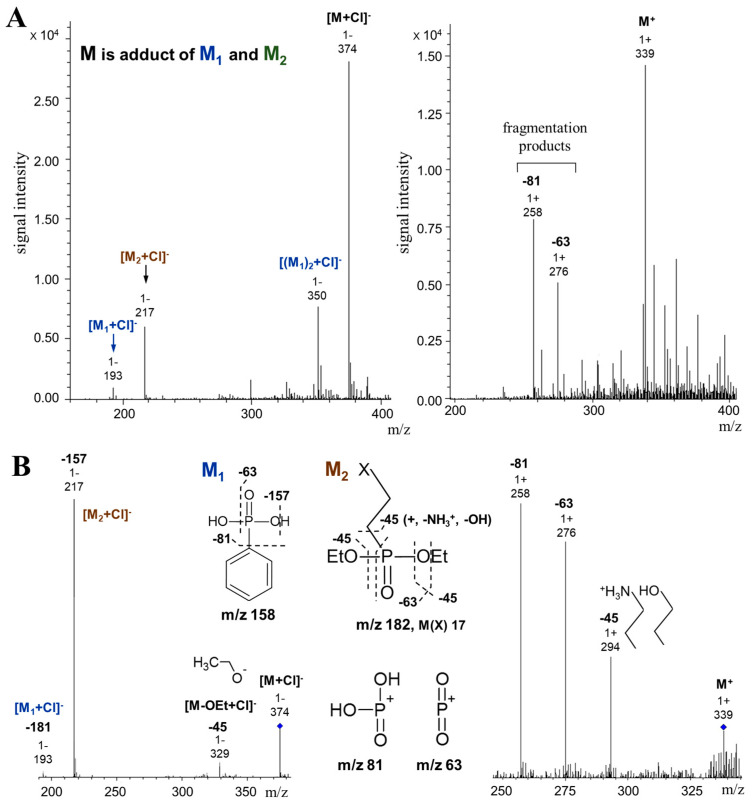
ESI-QTOF spectra of the phosphonate-containing fraction of the *Pectobacterium atrosepticum* cultural supernatant. (**A**) Mass spectra of the sample in negative and positive modes. (**B**) MS/MS spectra of major ions 374^1−^ and 339^1+^ and the scheme of their possible fragmentation.

The mass spectra of the sample in positive and negative modes revealed several signals with predominant ions 374^1−^ and 339^1+^ (Figure 4A). These ions had different fragmentation “behavior”. The ion with 374 *m*/*z* required higher collision energy for decay and produced predominantly two products—the major ion 217^1−^ (−157) and the minor one 329^1−^ (−45)—presumably representing the ethyl group (Figure 4B) also detected in the NMR spectra (Figure 3). Among the minor ions in this spectrum, signal 193^1−^ (−181) was also detected. Both the 193^1−^ and 217^1−^ ions were present in the initial MS spectrum of the sample, demonstrating their fragmentary origin from the 374^1−^ ion. Both ions were not fragmented. The difference in the mass of the 374^1−^ and 339^1+^ ions suggested the formation of a charge in the negative mode due to the addition of the Cl^−^ ion. The ionization of the target compound due to the addition of Cl^−^ ion ensured the high stability of the ions at fragmentation. The removal of Cl^−^ ions, and protonation of the products suggested that the 374^1−^ ion is an adduct of compounds with molecular weights of 158 and 182. This was consistent with the results of its fragmentation: the fragmentation spectrum of the 339^1+^ ion was represented by ions 258^1+^ (−81), 276^1+^ (−63), and 294^1+^ (−45). The most intense signals in the fragmentation spectrum of ion 339^1+^ (276^1+^ and 258^1+^) arized due to the removal of the phosphinate (−63) and the fragment of phosphonate (−81) groups, respectively. The 294^1+^ ion was formed due to the removal of a fragment with a molecular weight of 45, which was presumably related to the aliphatic fragment of the compound with 182 *m*/*z* (Figure 4B). The remaining product 294^1+^ represented an adduct of a fragment (ethylated phosphonate group, molecular weight 137) of compound M_2_ and compound M_1_. The ionization of the adduct in the positive mode was ensured by the formation of a charge on the P atom due to the breaking of the double bond during adduct formation. Thus, the NMR and mass spectrometry results suggested that the target sample contained two types of phosphonates with molecular weights of 158 and 181 or 182 (depending on the structure of the aliphatic fragment). Signals 193^1−^ and 217^1−^ represented adducts of these compounds with chlorine ions, respectively. The first-type phosphonate, in addition to the phosphonate group, contained a phenyl group, while the second one contained an aliphatic fragment with nonequivalent protons (a methylene group and, presumably, a hydroxyl group or amino group protonated in a positive mode).

**Figure 5:**
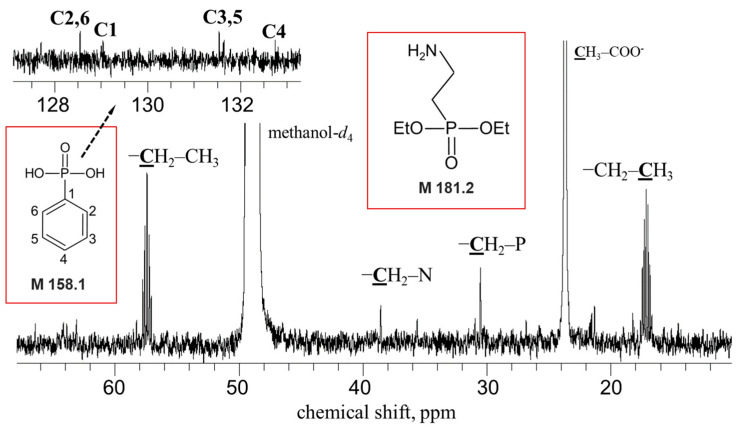
Fragments of the ^13^C spectrum of the phosphonate-containing fraction of the *Pectobacterium atrosepticum* cultural supernatant purified by chromatography and the molecular structures of the identified phosphonates (marked by red frames). EtO—ethyl group.

To clarify the structure of the aliphatic fragment of one of the revealed phosphonates, the initial sample was further purified using chromatography, tested for the presence of a phosphonate group using ^31^P NMR, and reanalyzed using ^13^C NMR. Of the seven fractions obtained after chromatographic separation, phosphonate signals were detected using ^31^P NMR in only one fraction (pH 4.5). In the ^13^C spectra of this fraction, signals at 17.2 and 57.4 ppm of comparable intensity (Figure 5) confirmed the presence of ethyl groups in the composition of the target phosphonate. The signals at 30.5 and 38.6 ppm were assigned to methylene groups bound to the phosphorus and nitrogen atoms, respectively. Thus, the collective results of NMR and mass spectroscopy showed that the structure of one of the target *Pba* phosphonates was represented by 2-diethoxyphosphorylethanamine (molecular weight of 181.2, protonated form of 182.2). Four signals of weak intensity in the weak field region (δ 127.2, δ 128.4, δ 130.9, and δ 131.4 ppm) in ^13^C spectra (Figure 5) indicated that the second *Pba* phosphonate was represented by phenylphosphonic acid (molecular weight of 158.1). The contribution of phenylphosphonic acid to the total pool of *Pba* phosphonates in both initial (Figure 4A) and purified (Figure 5) samples was significantly lower compared to 2-diethoxyphosphorylethanamine.

### 2.3. Regulation of the Production of Pba Phosphonates

The production of most (if not all) *Pba* virulence factors is controlled via quorum sensing (QS) and the perception of the products of the degradation of pectic compounds [26,27,28,29,30]. Therefore, we investigated whether QS and the presence of pectic compounds have a regulatory effect on the expression of the *fom1* gene and the production of phosphonates.

**Figure 6:**
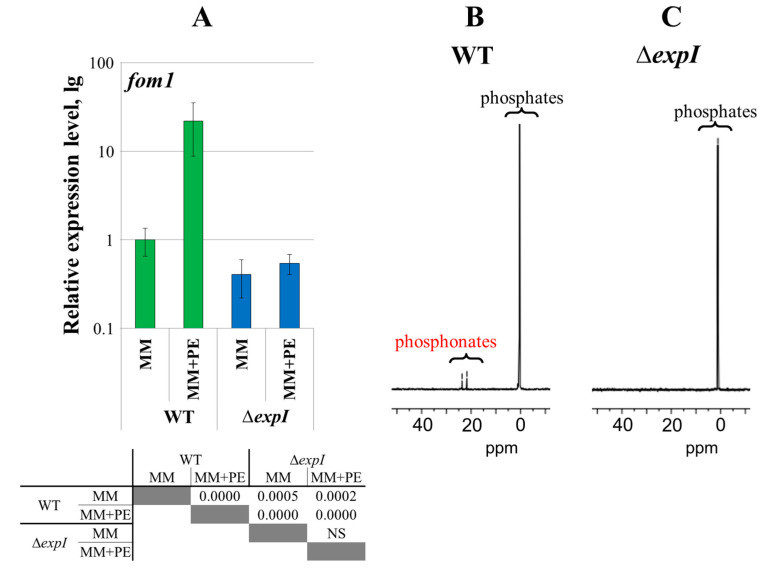
Effect of quorum sensing on phosphonate production in *Pectobacterium atrosepticum* SCRI1043. (**A**) Expression levels of the *fom1* gene in wild-type *P. atrosepticum* (WT) and *P. atrosepticum* quorum-deficient Δ*expI* mutant grown for 24 h in minimal medium (MM), and MM supplemented with plant extract (MM + PE). The expression level of the *fom1* gene in the WT cells grown in MM is equated to one. The presented values were obtained from five biological replicates. The table located under the diagram shows significant differences (*p*-values) between the designated experimental groups (Mann–Whitney two-sided test with Bonferroni correction for multiple comparisons, *p* < 0.05, five biological replicates). NS—non-significant. (**B**,**C**) ^31^P NMR spectra of the preparations of cultural supernatants of the wild-type *P. atrosepticum* (WT) (**B**) and *P. atrosepticum* Δ*expI* mutant (**C**) grown for 48 h in minimal medium with plant extract (MM + PE). The detection of phosphonates was performed in at least three independent experiments.

To analyze the effects of QS, a quorum-deficient Δ*expI* mutant [31] was used. The expression level of the *fom1* gene was 40-fold lower in the Δ*expI* mutant than in the WT under MM + PE conditions (Figure 6A). No ^31^P NMR signals at 15–25 ppm were detected in the cultural supernatants of the Δ*expI* mutant in contrast to those of the WT (Figure 6B,C).

**Figure 7:**
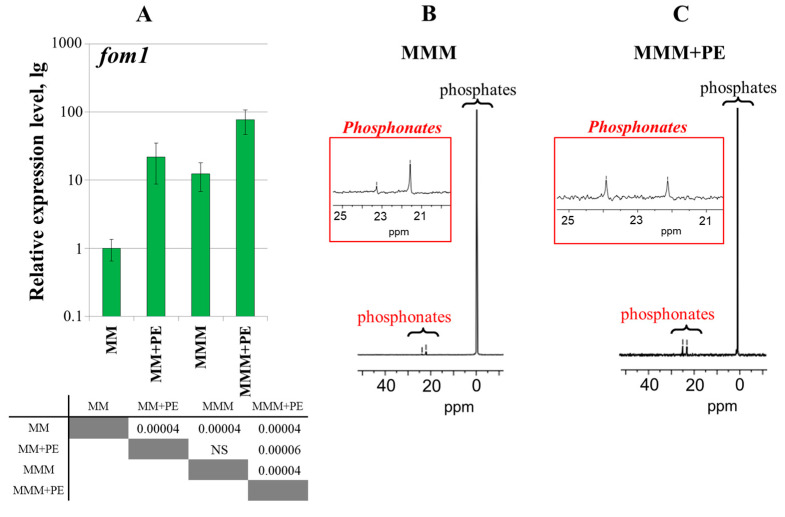
Effect of pectic compounds on phosphonate production in *Pectobacterium atrosepticum* SCRI1043. (**A**) The expression levels of the *fom1* gene in *P. atrosepticum* cells grown for 24 h in (1) minimal medium (MM), (2) MM supplemented with plant extract (MM + PE), (3) modified minimal medium where sucrose was substituted with pectin (MMM), (4) MMM supplemented with plant extract (MMM + PE). The expression level of the *fom1* gene in the cells grown in MM is equated to one. The presented values were obtained from five biological replicates. The table located under the diagram shows significant differences (*p*-values) between the designated experimental groups (Mann–Whitney two-sided test with Bonferroni correction for multiple comparisons, *p* < 0.05, five biological replicates). NS—non-significant. (**B**,**C**) ^31^P NMR spectra of the preparations of supernatants of *P. atrosepticum* cultures grown for 48 h in MMM (**B**) and MMM supplemented with plant extract (MMM + PE) (**C**). The detection of phosphonates was performed in at least three independent experiments.

To analyze the effect of pectic compounds on *Pba* phosphonate production, the WT strain was grown under four conditions: (1) MM, (2) MM + PE, (3) MMM (modified minimal medium where sucrose was substituted with pectin), (4) MMM + PE. The substitution of sucrose with pectin in the growth medium resulted in a 12-times increased expression level of the *fom1* gene (as well as supplementation of sucrose-containing medium with plant extract) (Figure 7A). The highest expression level of the *fom1* gene was observed in cells cultured in the presence of both pectic compounds and plant extract (MMM + PE) (Figure 7A). ^31^P NMR signals at 15–25 ppm were detected in the supernatants of cultures grown in pectin-containing medium, regardless of the presence of plant extract (Figure 7B,C).

### 2.4. Effect of Pba Phosphonates on Plant–Pba Interactions

To analyze the effects of *Pba* phosphonates on plant–*Pba* interactions, we assayed whether phosphonate deficiency affected *Pba* virulence. Tobacco plants were infected with either WT, Δ*fom1* mutant, or complemented Δ*fom1* mutant. No significant differences in virulence were observed between the three assayed strains, with 80–90% of plants displaying disease symptoms (tissue maceration) five days after infection in different experimental groups. Previously, we encountered a situation in which the knockout of virulence-related genes was not manifested under infection-promoting conditions but was manifested when virulence was assessed toward primed plants (with enhanced immune status) [32,33]. Therefore, we compared the virulence of the three assayed strains toward plants primed with 0.2 or 1.0 mM of salicylic acid (SA), which is known to promote resistance to *Pba* [12,32,34].

**Figure 8:**
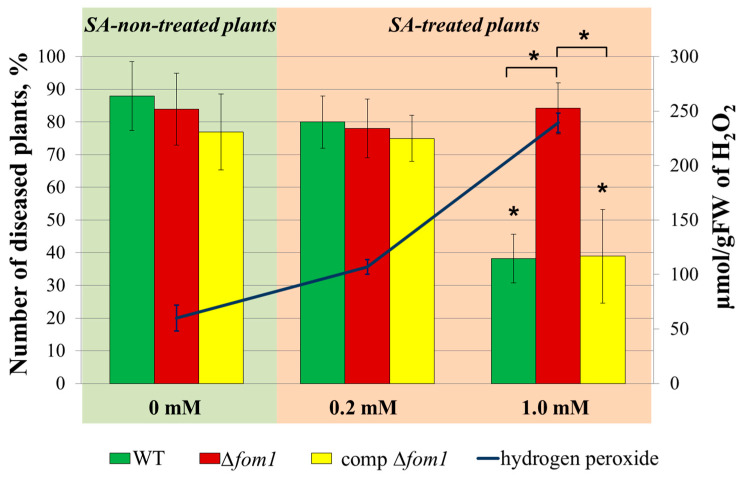
Virulence of the wild-type *Pectobacterium atrosepticum* SCRI1043 (WT) (green column), *P. atrosepticum* Δ*fom1* mutant (red column), and complemented Δ*fom1* mutant carrying the *fom1* gene within the recombinant plasmid (yellow column) toward tobacco plants non-treated (0 mM) with salicylic acid (SA) or primed with 0.2 or 1.0 mM of SA. Plants were pretreated with SA (or water) one day before infection. The percentage of plants with visible disease symptoms (tissue maceration) was assessed on the fifth day after infection. The presented results were obtained from 7 independent experiments; in each experiment, 20–25 plants were assessed for each experimental variant. Asterisks (*) show a significant difference (Mann–Whitney two-sided test, *p* < 0.05) from the variant where SA-non-treated plants were infected with the wild-type *P. atrosepticum* (first column); asterisks above the brackets show a significantly higher disease incidence rate in plants primed with 1.0 mM SA following infection with Δ*fom1* mutant than with WT or complemented Δ*fom1* mutant. The dark blue line shows the level of hydrogen peroxide in plants one day after treatment with 0, 0.2, or 1.0 mM of SA.

The treatment of plants with SA led to the concentration-dependent accumulation of hydrogen peroxide: 0 mM of SA—60 µmol/gFW of H_2_O_2_; 0.2 mM of SA—107 µmol/gFW of H_2_O_2_; and 1.0 mM of SA—240 µmol/gFW of H_2_O_2_ (Figure 8). All three strains were as virulent toward 0.2 mM SA-treated plants as they were toward SA-non-treated plants, and no difference in virulence was observed for the three strains (Figure 8). The virulence of WT and the complemented Δ*fom1* mutant toward 1.0 mM SA-treated plants was significantly reduced compared to the virulence toward SA-non-treated plants. Herewith, the Δ*fom1* mutant remained as virulent toward 1.0 mM SA-treated plants as it was toward SA-non-treated plants (Figure 8). Thus, the production of phosphonates by *Pba* appeared to have a negative effect on *Pba* virulence.

We considered it most likely that the negative effect of phosphonates on *Pba* virulence was related to their negative effects on either the resistance of *Pba* to H_2_O_2_ (that was accumulated in plants following the SA treatment) or the production of PCWDEs. To check these hypotheses, in all three strains, we compared resistance to H_2_O_2_ and extracellular pectate lyase, polygalacturonase, cellulase, and protease activities.

**Figure 9:**
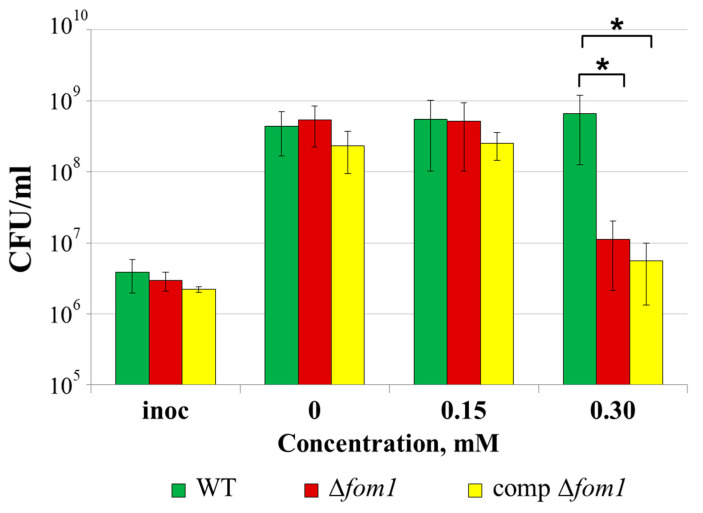
Resistance of the wild-type *Pectobacterium atrosepticum* SCRI1043 (WT) (green column), *P. atrosepticum* Δ*fom1* mutant (red column), and complemented Δ*fom1* mutant carrying the *fom1* gene within the recombinant plasmid (yellow column) to oxidative stress (H_2_O_2_). Cells were cultured in modified minimal medium supplemented with plant extract (MMM + PE) for 24 h in the presence of 0, 0.15, and 0.30 mM of H_2_O_2_ before plating for CFU titer analysis. The presented values are the means of five biological replicates. Inoc—inoculation titer. Asterisks (*) show the significance of the difference (Mann–Whitney two-sided test, *p* < 0.05) in cell titer between variants designated by brackets.

The presence of 0.15 or 0.30 mM of H_2_O_2_ did not affect the growth of the wild type in the modified minimal medium supplemented with plant extract (MMM + PE), whereas the growth of the Δ*fom1* mutant was reduced by two orders of magnitude in the presence of 0.30 mM of H_2_O_2_ (Figure 9). Unexpectedly, the complementation of the Δ*fom1* mutation did not restore the H_2_O_2_ resistance of the Δ*fom1* mutant to the wild-type level (Figure 9).

**Figure 10:**
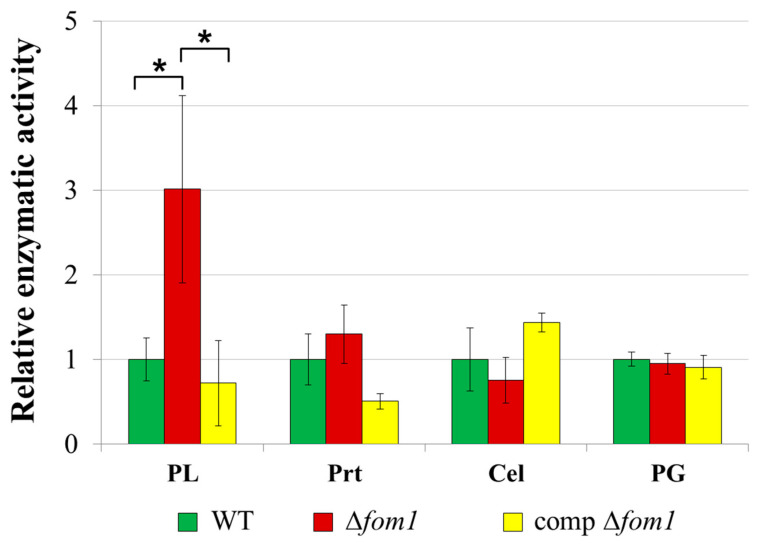
Relative levels of extracellular pectate lyase (PL), protease (Prt), cellulase (Cel), and polygalacturonase (PG) activities in the wild-type *Pectobacterium atrosepticum* SCRI1043 (WT) (green column), *P. atrosepticum* Δ*fom1* mutant (red column), and complemented Δ*fom1* mutant carrying the *fom1* gene within the recombinant plasmid (yellow column). Enzymatic activities were determined in the cultural supernatants after one day of cultivation of bacteria in the modified minimal medium supplemented with plant extract (MMM + PE). The presented values are the means of at least five biological replicates. The activity levels of the wild type were equated to one. Asterisks (*) show the significance of the difference (Mann–Whitney two-sided test, *p* < 0.05) between variants designated by brackets.

The knockout of the Δ*fom1* gene did not lead to alterations in extracellular polygalacturonase, cellulase, or protease activities (Figure 10). Herewith, pectate lyase activity increased three times in the mutant strain, whereas the complementation of the mutation led to a decrease in pectate lyase activity to the wild-type level (Figure 10).

Thus, *Pba* phosphonates have a positive effect on *Pba* stress (H_2_O_2_) resistance and a negative effect on extracellular pectate lyase activity.

## 3. Discussion

Given that *Pba* phosphonate-biosynthetic genes were among the most upregulated in planta vs. in vitro conditions [10], we tested our hypothesis of whether *Pba* indeed produces phosphonates and whether these compounds (if produced) play a role in plant–*Pba* interactions. We showed that *Pba* indeed produces LMW extracellular phosphonates and that the *fom1* gene (encoding the predicted phosphoenolpyruvate mutase, a crucial phosphonate-biosynthetic enzyme) determines the ability of *Pba* to synthesize these compounds (Figure 1 and Figure 2).

*Pba* synthesized two structural types of extracellular LMW phosphonates: 2-diethoxyphosphorylethanamine and phenylphosphonic acid (Figure 5). Among natural phosphonates, such structures have not previously been described; however, both 2-diethoxyphosphorylethanamine and phenylphosphonic acid have previously been obtained by chemical synthesis [35,36,37,38].

Among the phosphonates of phytopathogenic bacteria, the structures of only two compounds were determined before our study: pantaphos (2-(hydroxy[phosphono]methyl)maleate) produced by *Pantoea ananatis* [25] and fosfomycin ([(2R,3S)-3-methyloxiran-2-yl]phosphonic acid) produced by *Pseudomonas syringae* [22]. All four phosphonates described in phytopathogenic bacteria (produced by *P. ananatis*, *P. syringae* [22,25], and two produced by *Pba* (this study)) have significant distinctive features in their structures. In particular, *Pba* phosphonates, in contrast to pantaphos and fosfomycin, have either two ethyl groups and one amino group (one of the revealed compounds) or a phenyl group (another of the revealed compounds) (Figure 5). This presumably means that these four phosphonates implement different functions. The presence of an aromatic group within LMW phosphonates seems like a rather rare phenomenon; as far as we know, only phenyl-substituted LMW phosphonates have been described in *Actinomycetes* [39,40,41,42].

In our study, the production of *Pba* phosphonates (as well as the expression of the *fom1* gene) was induced in vitro by the presence of plant extract, which is in agreement with a previous study showing the upregulation of phosphonate-related genes in vitro following the addition of plant extract (Figure 1) [17]. Moreover, we showed that QS and the presence of pectic compounds have strong positive regulatory effects on the production of *Pba* phosphonates (Figure 6 and Figure 7). The positive effect of plant extract on the expression of the *fom1* gene is unlikely to be explained only by the presence of some pectic compounds in its composition, since plant extract positively affected the expression of the *fom1* gene even when pectic compounds constituted a major carbon source in the growth medium (Figure 7). This implies that, in addition to pectin, other plant-derived compounds have a positive effect on the production of *Pba* phosphonates.

The regulation of the production of microbial phosphonates is almost uninvestigated. To the best of our knowledge, only the effect of culture conditions on phosphonate production in several bacterial species (*Streptomyces* sp., *Bacillus* sp., *Saccharothrix* sp.) has been analyzed so far [43,44,45,46], but no regulatory systems have been previously described to control the production of phosphonates in any bacteria. Thus, our study provides the first insight into the mechanisms of the regulation of phosphonate biosynthesis.

In *Pantoea ananatis*, it was possible to achieve the production of detectable levels of phosphonate pantaphos only by genetic manipulation, namely by introducing the isopropyl thiogalactopyranoside (IPTG) promoter upstream of the phosphoenolpyruvate mutase gene [24]. In our study, the production of detectable levels of *Pba* phosphonates could be achieved by the addition of plant extract (as well as pectic compounds) to *Pba* cultures without any genetic manipulation (Figure 1 and Figure 7).

Since the production of *Pba* phosphonates (as well as most of *Pba* virulence factors [26,27,28,29]) was positively regulated by QS, as well as in the presence of plant extract and pectic compounds, we presumed that phosphonates are the virulence factors of *Pba*. This assumption was also inspired by a previous finding that *Pantoea ananatis* pantaphos has phytotoxic properties and determines *P. ananatis* virulence [24]. However, the knockout of the *fom1* gene that led to the inability of *Pba* to produce phosphonates did not affect *Pba* virulence (Figure 8).

It has previously been shown that the inability to synthesize one of many virulence factors may have no effect on virulence under conditions that promote disease development; herewith, under certain conditions, e.g., when the plant immune system is in primed status, the deficiency in this virulence factor is manifested in impaired virulence [32,33]. Therefore, we tested whether phosphonate deficiency affected *Pba* virulence in plants treated with SA, which led to the accumulation of H_2_O_2_ and increased plant resistance to *Pba* [12,32]. Intriguingly, SA-priming associated with the accumulation of H_2_O_2_ did not affect the virulence of the phosphonate-deficient mutant, although the virulence of the wild type (as well as the complemented mutant) was significantly reduced toward the SA-primed plants compared to virulence toward non-primed plants (Figure 8). This means that the production of phosphonates has a negative effect on *Pba* virulence.

Then, we attempted to understand how phosphonates could reduce *Pba* virulence and presumed that this could be related to their negative effects on either *Pba* resistance to H_2_O_2_ (which was accumulated in the SA-primed plants) or *Pba* PCWDE activities, or both. However, instead of the expected negative effect of phosphonates on *Pba* H_2_O_2_ resistance, we found that phosphonate deficiency resulted in reduced *Pba* H_2_O_2_ resistance (Figure 9). This means that phosphonates contribute to *Pba* stress resistance. However, phosphonate-deficiency-mediated compromised H_2_O_2_ resistance was not only unrelated to reduced *Pba* virulence but was even associated with improved virulence toward plants with an increased H_2_O_2_ level. This likely means that phosphonates might have such a significant negative effect on some virulence-associated traits that phosphonate deficiency could cause increased virulence even in the background of reduced H_2_O_2_ resistance.

Indeed, we found that the production of phosphonates negatively affected the activity of extracellular pectate lyases, which are considered the main virulence factors of pectolytic bacteria. Herewith, no effect of phosphonates was revealed with regard to the activities of other *Pba* PCWDEs (polygalacturonase, protease, and cellulase) (Figure 10).

The negative effect of phosphonates on the activity of different enzymes is rather well described in the framework of their antibiotic or herbicidal action [18,19,20]. Phosphonates by direct binding with the particular enzyme can repress its activity or prevent enzyme–substrate interaction [47,48]. Whether phosphonates can regulate the production of enzymes at transcriptional or translational levels is not yet understood. In our further studies, we will tackle the question of the mechanisms by which *Pba* phosphonates reduce pectate lyase activity. We will also try to understand whether the revealed effect of *Pba* phosphonates on pectate lyase activity is direct or mediated by their influence on other physiological processes, as well as whether the assayed metabolites cause other effects on *Pba* or host plant physiology.

In addition, given that two types of *Pba* phosphonates were revealed in our study, we cannot currently conclude which of them or whether both of them together are responsible for the observed phenotypes. For example, it is possible that one of the revealed compounds promotes an increased *Pba* H_2_O_2_ resistance, while the other one is responsible for the reduced *Pba* virulence, or, in contrast, both compounds together regulate an ensemble of physiological processes that are expressed in increased H_2_O_2_ resistance coupled with relatively modest virulence. The clarification of the particular roles of each particular *Pba* phosphonate is currently complicated due to the fact that only the “root” part of the phosphonate-biosynthetic pathway is predicted in *Pba*. Therefore, in our study, to analyze the phenotypic manifestation of phosphonate deficiency, we could only obtain the mutant that is deficient in both of the revealed phosphonates. Further deciphering of the phosphonate-biosynthetic pathway will form a basis for obtaining and analyzing mutants deficient in one of the two revealed phosphonates to gain deeper insight into the physiological functions of each separate phosphonate. Nevertheless, our study shows that the revealed *Pba* phosphonates (either the first one, the second one, or both together) exert a rather global physiological effect on *Pba*, positively controlling stress resistance and negatively controlling virulence.

It is well accepted that the production of virulence factors in plant pathogenic bacteria is negatively controlled by a number of regulatory proteins to prevent unnecessary (or even harmful) synthesis of virulence factors outside of the host or at early infection stages [49]. However, we did not find any information on phytopathogen-produced LMW compounds that repressed phytopathogens’ own PCWDEs. The only similar phenomenon has been previously described in *Xylella fastidiosa*. In this species, specific LMW mediators of QS (diffusible signal factor, DSF) repress biofilm formation [50,51], whereas in most (if not all) of the other species, QS mediators positively affect biofilm formation [52,53,54]. Such an “unconventional” situation with *X. fastidiosa* was explained by the fact that this bacterium is a xylem-limited phytopathogen, and therefore, although biofilm formation is required for vessel colonization, the extensive blockage of the vessel by too large biofilm would reduce the influx of nutrients toward the pathogen and cause rapid death of the host, both of which are harmful to the pathogen [50]. To prevent these negative consequences of extensive vessel colonization, DSF, by repressing virulence-associated traits at rather advanced stages of plant–microbe interaction, enables the pathogen to control its “harmful properties”, and thus prolongs the functioning of the pathosystem.

The discovered *Pba* phosphonates presumably also enable *Pba* to control its “harmful properties” and restrain the activity of the main virulence factors (and maybe some other virulence-associated traits), maintaining its ecological niche (the host plant) in a more or less functional state for a longer period of time. It is reasonable to speculate that for most (if not all) phytopathogens, it is important to be able to produce metabolites that reduce their pathogenic potential when necessary to ensure durable plant–microbe interactions. The discovery of these metabolites and then the finding of approaches for manipulating microbial behavior through these metabolites seem like a good strategy for reducing damage caused by phytopathogens in agriculture.

## 4. Materials and Methods

### 4.1. Bacterial Strains, Media, and Culture Conditions

*Pectobacterium atrosepticum* SCRI1043 (*Pba*) (ATCC BAA-672) was grown in lysogeny broth (LB) medium on a rotary shaker (180 rpm) at 28 °C. The Δ*fom1* mutant and Δ*expI* mutant strains were grown in the presence of kanamycin (20 μg/mL); the complementation Δ*fom1* mutant was grown in the presence of kanamycin (20 μg/mL) and ampicillin (200 μg/mL). The CFU titer was determined by plating serial 10-fold dilutions of the cultures onto 1.5% LB agar.

In the framework of experimental models described in the Result section (Section 2), bacteria were cultured at 180 rpm and 28 °C in four media: (1) minimal medium (MM), containing 50 mM of potassium phosphate buffer, 7.6 mM of (NH_4_)_2_SO_4_, 1.7 mM of MgCl_2_, 1.7 mM of NaCl, and 2.0 g/L of sucrose, with a pH of 7.0 [55]; (2) MM supplemented with 1/10 (*v*/*v*) of plant extract (MM + PE); (3) modified minimal medium (MMM) that contained the same components as MM, except that sucrose was substituted with 2.0 g/L pectin (FLUKA Biochemika, Buchs, Switzerland); (4) MMM supplemented with 1/10 (*v*/*v*) of plant extract (MMM + PE).

The plant extract was prepared by grinding 500 g of potato tubers in 1 L of water in a blender. The mixture was centrifuged at 8000× *g* for 20 min. The supernatant was then heated to 100 °C, cooled to room temperature, and centrifuged at 8000× *g* for 20 min. The supernatant was further clarified by filtering through 0.45 μm and 0.22 μm nitrocellulose filters (Corning, Berlin, Germany). Finally, the extract was filter sterilized through 0.22 μm nitrocellulose filters and stored at −20 °C.

### 4.2. Gene Expression Analysis

For the isolation of total RNA, bacterial cells grown for one day in LB, MM, MM + PE, MMM, and MMM + PE were harvested at 8000× *g* for 5 min. Bacterial cell pellets were resuspended in 1 mL of ExtractRNA Reagent (Evrogen, Moscow, Russia), and the subsequent procedures were performed according to the manufacturer’s instructions. RNA samples were treated with DNAse I using a DNA-free kit (Thermo Fisher Scientific, Waltham, MA, USA). RNA quantity and quality were analyzed using a NanoPhotometer NP80 (Implen, Munchen, Germany) and electrophoresis in a 1% agarose gel, respectively. One microgram of DNAse-treated RNA was used for cDNA synthesis using RevertAid reverse transcriptase (Thermo Fisher Scientific, Waltham, MA, USA) according to the manufacturer’s instructions. Two microliters of 5-fold-diluted cDNA preparation were used for qPCR. qPCR was performed using the EvaGreen-containing master mix (Syntol, Moscow, Russia) according to the manufacturer’s instructions. Primers for target (*fom1*) and reference genes (Table S1) were designed using Vector-NTI Version 9 software (Invitrogen, Carlsbad, CA, USA) and synthesized by Evrogen (Moscow, Russia).

PCR was performed under the following conditions: 95 °C for 2 min, followed by 45 cycles at 94 °C for 10 s, 60 °C for 15 s, and 72 °C for 30 s. After that, melt curve analysis was performed in the temperature range of 60 to 95 °C. The reactions were run and changes in fluorescence emission were detected using a CFX96 quantitative PCR system (Bio-Rad, Hercules, CA, USA). The amount of fluorescence was plotted as a function of the PCR cycle using CFX Manager Software Version 2.1 (Bio-Rad, Hercules, CA, USA). The amplification efficiency (E) for all primers was determined using a dilution series of a pool of cDNAs. The additional controls included the omission of reverse transcriptase to measure the extent of residual genomic DNA contamination and the omission of a template.

Genes encoding RNA polymerase sigma factor RpoD (ECA0680), signal recognition particle protein Ffh (ECA3360), and protein RecA (ECA3369), the transcript levels of which were confirmed by geNorm software v. 3.5 (http://genorm.cmgg.be, accessed on 20 May 2023) to be stable under the experimental conditions, and were used for normalization of the target gene expression. Relative expression levels were determined as the ratios between the quantities of cDNA corresponding to the target genes and values of the normalization factor, which were calculated for each sample using geNorm software based on the transcript levels of reference genes. The presented data were obtained by analyzing 5 biological replicates.

### 4.3. Detection of Extracellular Phosphonates Using ^31^P NMR

For the detection of extracellular phosphonates, bacterial cells were grown for two days in MM, MM + PE, MMM, and MMM + PE media at 28 °C and 180 rpm. Cultural supernatants were separated from bacterial cells by centrifugation at 8000× *g* for 20 min and subsequent filtration of the supernatants through 0.22 μm nitrocellulose filters (Corning, Berlin, Germany). The filtered supernatants (500 mL) were concentrated in an Alpha 2–4 LD plus freeze dryer (Christ, Frankfurt am Main, Germany) to 10–50 mL. Then, methanol was added to a concentration of 75%, and samples were incubated overnight at −20 °C. Insoluble material was removed by centrifugation (10,000× *g*, 20 min), and the supernatant was concentrated by rotary evaporation to 5 mL. Then, methanol was added to a concentration of 90%, and samples were incubated overnight at −20 °C. The insoluble material was removed by centrifugation (10,000× *g*, 20 min), and the supernatant was concentrated by rotary evaporation to dryness [56]. The precipitate was dissolved in 0.5 mL of distilled water. ^31^P NMR spectra were recorded on a Bruker Avance II-400 (working frequency 161.9 MHz, Bruker, Karlsruhe, Germany) spectrometer using 85% H_3_PO_4_ as an external standard.

### 4.4. Construction of a fom1 Deletion Mutant

The *fom1* deletion mutant (Δ*fom1*) was constructed by the method described by Kaniga et al. [57]. The target gene *fom1* (ECA0487 locus) together with adjacent regions (approximately 1000 bp up- and downstream of the *fom1* ORF) was amplified by PCR with the primers up*fom1*F and dn*fom1*R (Table S1) using Q5 high-fidelity DNA polymerase (NEB, Ipswich, MA, USA). The amplified PCR fragment was cloned into the bacterial cloning vector system pGEM-T Easy (Promega, Madison, WI, USA). The obtained plasmid (pGEM:*fom1*) was introduced into *E. coli* NovaBlue by chemical transformation. Transformants carrying the recombinant plasmid were screened for ampicillin resistance and further verified by PCR using plasmid-specific primers for the T7 and SP6 polymerase promoters, which flank the multiple cloning regions of pGEM-T Easy.

To replace the *fom1* ORF with the Km^R^ cassette, a part of the pGEM:*fom1* plasmid (including ~1000 bp regions up- and downstream of *fom1* ORF, but not *fom1* ORF itself) was amplified using the primers dn*fom1*KmF and up*fom1*KmR (Table S1), whose 5′ ends were complementary to the end regions of the Km^R^ cassette. The amplified PCR fragment was treated with restriction endonuclease DpnI to remove the original methylated plasmid and then purified using a DNA cleanup kit (NEB, Ipswich, MA, USA). The Km^R^ cassette was amplified from the pKD4 plasmid using the primers Km*fom1*F and Km*fom1*R (Table S1), whose 5′ ends were complementary to the *Pba* DNA regions adjacent to the *fom1* ORF. Two obtained PCR fragments (corresponding to the pGEM plasmid with ~1000 bp regions up- and downstream of the *fom1* ORF and to the Km^R^ cassette) were joined by the circular polymerase extension cloning method [58]. The obtained plasmid (pGEM:Δ*fom1*;Km^R^) was introduced into *E. coli* NovaBlue by chemical transformation. The mutant locus was confirmed by DNA sequencing.

The mutant locus (containing the Km^R^ cassette and ~1000 bp regions up- and downstream of *fom1* ORF) was amplified with the primers up*fom1*F and dn*fom1*R (Table S1) and ligated (T4 ligase, NEB, Ipswich, MA, USA) into the SmaI-digested (NEB, Ipswich, MA, USA) suicide vector pKNG101 to generate a recombinant plasmid containing the allelic exchange cassette for the target locus. The obtained plasmid (pKNG101:Δ*fom1*;Km^R^) was introduced into *E. coli* cc118 by electroporation. The transfer of the pKNG101:Δ*fom1*;Km^R^ plasmid from *E. coli* cc118 into *Pba* was achieved by triparental mating using *E. coli* HH26 as a helper strain. Clones in which the pKNG101:Δ*fom1*;Km^R^ plasmid was integrated into the chromosome by a single crossover event were selected by streptomycin and kanamycin resistance. Clones in which the second crossover event led to the replacement of the target locus with the mutant one and the donor plasmid was eliminated were selected on an M9 agar medium containing 10% sucrose. Then, the clones were tested for streptomycin sensitivity. Clones without streptomycin resistance were analyzed by PCR using the primers Check*fom1*F and Check*fom1*R (Table S1) to identify Δ*fom1* mutants.

### 4.5. Construction of the Δfom1 Complemented Mutant Strain Carrying the Fom1 Gene on the Plasmid

To construct the complementing plasmid, the 1443 bp region containing the target gene *fom1* with promoter and terminator regions was amplified by PCR with primers comp*fom1*F and comp*fom1*R (Table S1) using Q5 high-fidelity DNA polymerase (NEB, Ipswich, MA, USA). The amplified PCR fragment was cloned into the bacterial cloning vector system pGEM-T Easy (Promega, Madison, WI, USA). The obtained plasmid (pGEM:*fom1*;Amp^R^; the complementation construct) was introduced into *E. coli* NovaBlue by chemical transformation. Transformants carrying the recombinant plasmid were screened for ampicillin resistance and further verified by PCR using plasmid-specific primers for the T7 and SP6 polymerase promoters. The correctness of the assembly was confirmed by DNA sequencing.

The obtained plasmid, pGEM:*fom1*;Amp^R^ was introduced into the *Pba* Δ*fom1* (Km^+^) mutant by electroporation. Clones with ampicillin and kanamycin resistance were analyzed by PCR using the primers Km*fom1*F/Km*fom1*R and *fom1*F/*fom1*R (Table S1) to confirm the presence of the complementing plasmid.

### 4.6. Determination of the Molecular Structure of the Pba Phosphonates

The residue of the methanol-extractable fraction of the *Pba* cultural supernatants (obtained as described in Section 2.3) was dissolved in 50 mL of deionized water. NH_4_OH was added to the solution to yield a pH of 10. Then, 5 mL of 1 M calcium acetate was added to precipitate the phosphates. The precipitate was removed by centrifugation (10,000× *g*, 20 min). The remaining calcium was removed from the supernatant by the addition of 5 mL of 1M NH_4_HCO_3_ and centrifugation (10,000× *g*, 20 min). The supernatant was collected, methanol was added to yield a final concentration of 70%, and samples were incubated overnight at −20 °C. Then, the precipitate was removed by centrifugation (10,000× *g*, 20 min), and the supernatant was evaporated to dryness using rotary evaporation [59]. The fraction obtained after phosphate removal was used for NMR and mass spectrometric analysis. The presence of phosphonates in the samples was confirmed by the presence of signals at 15–25 ppm in the ^31^P NMR spectra.

To clarify the structural elements of the target phosphonates, the sample was further purified using ion-exchange chromatography. For this purpose, the sediment after phosphate removal was dissolved in 500 µL of deionized water and fractionated by 20 mM trifluoroacetic acid with aqueous ammonia (to obtain the required pH) on a column (1 × 40 cm) of Dowex 1-X8 (50–100 mesh; H_3_C-COO^−^ form (Merck KGaA, Darmstadt, Germany)) using a step gradient from pH 7.0 to 1.5. Fractions eluted at pH 7.0, 4.5, 4.3, 3.1, 2.8, 2.0, and 1.5 were collected and analyzed by ^31^P NMR. The fraction for which signals at 15–25 ppm were detected on the ^31^P NMR spectrum was analyzed by ^13^C NMR spectroscopy.

For ^1^H and ^13^C NMR analysis, samples were dissolved in D_2_O (99.9%, Ferak, Berlin, Germany) to accomplish the H-D exchange and re-dissolved after drying in D_2_O (99.994%, Aldrich, Burlington, MA, USA). The NMR spectra were recorded on a Bruker AVANCE II TM-500 NMR spectrometer operating at 500.1 MHz for ^1^H and 125.8 MHz for the ^13^C resonance, respectively. The spectra were recorded at 300 K. Methanol-*d*_4_ (δ 3.31 and 49.15 ppm) was used as an internal standard. Data processing and analysis were performed using Topspin 3.6.1 software (Bruker, Karlsruhe, Germany).

For mass-spectrometric analysis, the sample was dissolved in deionized water, and after the addition of an equal volume of methanol, it was injected by electrospray at atmospheric pressure (API-ESI). Spectra were recorded on a Bruker Daltonics MicrOTOF Q mass spectrometer (Bruker, Karlsruhe, Germany) in negative and positive modes; the rate of injection was 3 μL/min. Argon gas was used as the collision gas. High-purity nitrogen gas was used as the nebulizer and dry gas at a flow rate of 4 L/min. Collision energy in MS/MS analysis was −15 eV in negative mode and +12 eV in positive mode. The ESI source conditions were as follows: capillary 4100 V, end plate offset −500 V, capillary exit voltage 170 V, and dry gas temperature 200 °C. Data processing and analysis were performed using Bruker Daltonics DataAnalysis 3.4.

### 4.7. Plant Cultivation, Infection, and Priming

Tobacco plants (*Nicotiana tabacum* cv. Petit Havana SR1) were grown axenically in test tubes placed in a growth chamber with a 16 h light/8 h dark cycle photoperiod. Seeds were surface sterilized using diluted bleach (0.8% of active chlorine) and 1% sodium dodecyl sulfate for 30 min, washed seven times with sterile distilled water, and then transferred to Murashige and Skoog medium (MS) in Petri dishes. Ten-day-old seedlings were transferred to individual flasks containing MS. Four to five weeks after planting, plants with 5–6 leaves were infected with *Pba*, or Δ*fom1* mutant, or a complemented mutant. For plant inoculation, bacteria were grown until the early stationary phase (~2 × 10^9^ colony-forming units, CFU/mL), then washed with sterile 10 mM MgSO_4_ and resuspended in the same solution up to a density of ~2 × 10^7^ CFU/mL. Sterile 10 mM MgSO_4_ or bacterial suspensions containing ~2 × 10^5^ cells were placed as 10 μL drops on the leaf surface using sterile pipette tips, and, simultaneously, slight scratches were made at the inoculation point by touching it with the pipette tip. For plant priming, each plant was sprayed with ~350 µL of water (control) or 0.2 mM or 1.0 mM of salicylic acid 24 h before infection with bacteria. Symptoms were scored visually based on the presence or absence of visible macerated zones 1–5 days after infection. The presented results on plant infection were obtained from five to seven independent experiments; in each experiment, 20–25 plants were assessed for each experimental variant.

### 4.8. Determination of the Hydrogen Peroxide Level

H_2_O_2_ levels in tobacco leaves were measured 1 day after plant treatment with water or salicylic acid (0.2 mM or 1.0 mM). H_2_O_2_ levels were determined by a method based on the peroxide-mediated oxidation of Fe^2+^, followed by the reaction of Fe^3+^ with xylenol orange (Sigma, Ronkonkoma, NY, USA) [60]. Leaves (100 mg) were ground in 1 mL of cold 50 mM borate buffer (pH 8.4) in mortars. The homogenates were centrifuged (7000× *g*, 10 min), and 100 μL of the supernatants were added to 500 μL of the assay reagent (500 mM of ammonium ferrous sulfate, 50 of mM H_2_SO_4_, 200 of mM xylenol orange, and 200 of mM sorbitol). The absorbance of the Fe^3+^–xylenol orange complex (*A*560) was detected after 45 min using a microplate reader CLARIOstar (BMG Labtech GmbH, Ortenberg, Germany). Standard curves were obtained by adding various amounts of H_2_O_2_ to 100 μL of borate buffer mixed with 500 μL of the assay reagent. The data were normalized and expressed as µmol H_2_O_2_ per gram of fresh weight. The presented data are the means ± SD of three biological replicates.

### 4.9. Stress Tolerance Assay

To compare the H_2_O_2_ resistance of wild-type *Pba*, Δ*fom1* mutant, and complemented Δ*fom1* mutant, bacteria were cultured in a minimal medium supplemented with 1/10 of plant extract (MM + PE) in the absence or the presence of 0.15 or 0.3 mM of H_2_O_2_. After 24 h of cultivation, suspensions were plated onto 1.5% LB agar as serial 10-fold dilutions and incubated at 28 °C for 2 days before the CFUs were counted.

### 4.10. Enzymatic Activity Assays

Pectate lyase activity was determined by measuring the degradation of polygalacturonic acid (PGA) into unsaturated products [61]. First, 435 μL of 0.25% PGA (Sigma, Ronkonkoma, NY, USA) in 50 mM of Tris-HCl buffer (pH 8.5) was mixed with 50 μL of 10 mM CaCl_2_ and 50 μL of the cultural supernatant at 37 °C. The accumulation of unsaturated products was measured at 234 nm. One unit of pectate lyase activity was defined as the amount of enzyme releasing 1 μmol of unsaturated products/min per 10^9^ bacterial cells.

Cellulase and polygalacturonase activities were determined by measuring the reducing sugars released after the enzymatic hydrolysis of the corresponding substrates. The reducing sugars were measured using 3,5-dinitrosalicylic acid (DNS reagent) (Sigma, Ronkonkoma, NY, USA) at 540 nm [62]. Cellulase (endoglucanase) activity was determined using carboxymethyl cellulose as a substrate (Sigma, Ronkonkoma, NY, USA). Herewith, 250 μL of the cultural supernatant was mixed with 250 μL of 2% carboxymethyl cellulose in 100 mM citrate buffer (pH 5.5) and incubated for 30 min at 50 °C [63]. For the determination of polygalacturonase activity, 180 μL of PGA (5 mg/mL) in 50 mM of sodium acetate buffer pH 5.0 were mixed with 20 μL of bacterial supernatant and incubated for 60 min at 37 °C [64]. The reactions were stopped by heating at 100 °C for 5 min before the analysis of products by DNS reagent. One unit (U) of activities (cellulase and polygalacturonase) was defined as the amount of enzyme releasing 1 μmol of reducing sugars/min per 10^9^ bacterial cells.

For the protease activity assay, 500 μL of 1% azocasein in 100 mM Tris HCl (pH 7.5) and 100 μL of culture supernatant were mixed and incubated for 60 min at 37 °C, and then 100 μL of 10% trichloroacetic acid was added to the reaction mixture. The sediment was removed by filtration, 500 μL of the supernatant was incubated with 166 μL of 1M NaOH for 10 min at 25 °C, and the absorbance was measured at 440 nm [65]. One unit (U) of protease activity was defined as the amount of enzyme required to produce an absorbance change of 1.0/min per 10^9^ bacterial cells. All enzymatic activities were measured in at least five biological replicates using a CLARIOstar microplate reader (BMG Labtech GmbH, Ortenberg, Germany).

## Data Availability

The original contributions presented in this study are included in the article/Supplementary Materials. Further inquiries can be directed to the corresponding author.

## Supplementary Materials

The supporting information can be downloaded at: https://www.mdpi.com/article/10.3390/ijms252111516/s1. References [31,57,66,67,68,69] are cited in the supplementary materials.

## References

[1] Mansfield J., Genin S., Magori S., Citovsky V., Sriariyanum M., Ronald P., Dow M., Verdier V., Beer S.V., Machado M.A. (2012). Top 10 plant pathogenic bacteria in molecular plant pathology. Mol. Plant Pathol..

[2] Van Gijsegem F., van der Wolf J.M., Toth I.K., Van Gijsegem F., van der Wolf J.M., Toth I.K. (2021). Plant Diseases Caused by Dickeya and Pectobacterium Species.

[3] Charkowski A., Blanco C., Condemine G., Expert D., Franza T., Hayes C., Hugouvieux-Cotte-Pattat N., Solanilla E.L., Low D., Moleleki L. (2012). The role of secretion systems and small molecules in Soft-Rot *Enterobacteriaceae* pathogenicity. Annu. Rev. Phytopathol..

[4] Van Gijsegem F., Hugouvieux-Cotte-Pattat N., Kraepiel Y., Lojkowska E., Moleleki L.N., Gorshkov V.A., Yedidia I. (2021). Molecular interactions of *Pectobacterium* and *Dickeya* with plants. Plant Diseases Caused by Dickeya and Pectobacterium Species.

[5] Ageichik A.V., Evtushenkov A.N., Nikolaichik Y.A. (2002). The role of type III secretion system in *Erwinia carotovora* subsp. atroseptica virulence. Plant Prot. Sci..

[6] Holeva M.C., Bell K.S., Hyman L.J., Avrova A.O., Whisson S.C., Birch P.R.J., Toth I.K. (2004). Use of a pooled transposon mutation grid to demonstrate roles in disease development for *Erwinia carotovora* subsp. *atroseptica* putative type III secreted effector (DspE/A) and helper (HrpN) proteins. Mol. Plant-Microbe Interact..

[7] Rojas C.M., Ham J.H., Deng W.-L., Doyle J.J., Collmer A. (2002). HecA, a member of a class of adhesins produced by diverse pathogenic bacteria, contributes to the attachment, aggregation, epidermal cell killing, and virulence phenotypes of *Erwinia chrysanthemi* EC16 on *Nicotiana clevelandii* seedlings. Proc. Natl. Acad. Sci. USA.

[8] Mattinen L., Nissinen R., Riipi T., Kalkkinen N., Pirhonen M. (2007). Host-extract induced changes in the secretome of the plant pathogenic bacterium *Pectobacterium atrosepticum*. Proteomics.

[9] Liu H., Coulthurst S.J., Pritchard L., Hedley P.E., Ravensdale M., Humphris S., Burr T., Takle G., Brurberg M.-B., Birch P.R.J. (2008). Quorum sensing coordinates brute force and stealth modes of infection in the plant pathogen *Pectobacterium atrosepticum*. PLoS Pathog..

[10] Gorshkov V., Gubaev R., Petrova O., Daminova A., Gogoleva N., Ageeva M., Parfirova O., Prokchorchik M., Nikolaichik Y., Gogolev Y. (2018). Transcriptome profiling helps to identify potential and true molecular switches of stealth to brute force behavior in *Pectobacterium atrosepticum* during systemic colonization of tobacco plants. Eur. J. Plant Pathol..

[11] Gorshkov V.Y., Toporkova Y.Y., Tsers I.D., Smirnova E.O., Ogorodnikova A.V., Gogoleva N.E., Parfirova O.I., Petrova O.E., Gogolev Y.V. (2022). Differential modulation of the lipoxygenase cascade during typical and latent *Pectobacterium atrosepticum* infections. Ann. Bot..

[12] Tsers I., Parfirova O., Moruzhenkova V., Petrova O., Gogoleva N., Vorob’ev V., Gogolev Y., Gorshkov V. (2023). A Switch from latent to typical infection during *Pectobacterium atrosepticum*—Tobacco interactions: Predicted and true molecular players. IJMS.

[13] Enard C., Diolez A., Expert D. (1988). Systemic virulence of *Erwinia chrysanthemi* 3937 requires a functional iron assimilation system. J. Bacteriol..

[14] Franza T., Mahé B., Expert D. (2004). *Erwinia chrysanthemi* requires a second iron transport route dependent of the siderophore achromobactin for extracellular growth and plant infection. Mol. Microbiol..

[15] Dellagi A., Segond D., Rigault M., Fagard M., Simon C., Saindrenan P., Expert D. (2009). Microbial siderophores exert a subtle role in arabidopsis during infection by manipulating the immune response and the iron status. Plant Physiol..

[16] Islamov B., Petrova O., Mikshina P., Kadyirov A., Vorob’ev V., Gogolev Y., Gorshkov V. (2021). The role of *Pectobacterium atrosepticum* exopolysaccharides in plant-pathogen interactions. Int. J. Mol. Sci..

[17] Mattinen L., Somervuo P., Nykyri J., Nissinen R., Kouvonen P., Corthals G., Auvinen P., Aittamaa M., Valkonen J.P.T., Pirhonen M. (2008). Microarray profiling of host-extract-induced genes and characterization of the type VI secretion cluster in the potato pathogen *Pectobacterium atrosepticum*. Microbiology.

[18] Horsman G.P., Zechel D.L. (2017). Phosphonate biochemistry. Chem. Rev..

[19] Kafarski P., Churchill D., Dutour Sikirić M., Čolović B., Füredi Milhofer H. (2020). Phosphonates: Their natural occurrence and physiological role. Contemporary Topics About Phosphorus in Biology and Materials.

[20] Metcalf W.W., Van Der Donk W.A. (2009). Biosynthesis of phosphonic and phosphinic acid natural products. Annu. Rev. Biochem..

[21] Ju K.-S., Gao J., Doroghazi J.R., Wang K.-K.A., Thibodeaux C.J., Li S., Metzger E., Fudala J., Su J., Zhang J.K. (2015). Discovery of phosphonic acid natural products by mining the genomes of 10,000 Actinomycetes. Proc. Natl. Acad. Sci. USA.

[22] Shoji J., Kato T., Hinoo H., Hattori T., Hirooka K., Matsumoto K., Tanimoto T., Kondo E. (1986). Production of fosfomycin (phosphonomycin) by *Pseudomonas syringae*. J. Antibiot..

[23] García P., Arca P., Evaristo Suárez J. (1995). Product of *fosC*, a gene from *Pseudomonas syringae*, mediates fosfomycin resistance by using ATP as cosubstrate. Antimicrob. Agents Chemother..

[24] Asselin J.A.E., Bonasera J.M., Beer S.V. (2018). Center rot of onion (*Allium cepa*) caused by *Pantoea ananatis* requires *pepM*, a predicted phosphonate-related gene. Mol. Plant Microbe Interact..

[25] Polidore A.L.A., Furiassi L., Hergenrother P.J., Metcalf W.W. (2021). A Phosphonate natural product made by *Pantoea ananatis* is necessary and sufficient for the hallmark lesions of onion center rot. mBio.

[26] Barnard A.M.L., Salmond G.P.C. (2007). Quorum sensing in *Erwinia* species. Anal. Bioanal. Chem..

[27] Crépin A., Barbey C., Beury-Cirou A., Hélias V., Taupin L., Reverchon S., Nasser W., Faure D., Dufour A., Orange N. (2012). Quorum sensing signaling molecules produced by reference and emerging Soft-Rot Bacteria (*Dickeya* and *Pectobacterium* Spp.). PLoS ONE.

[28] Liu Y., Jiang G., Cui Y., Mukherjee A., Ma W.L., Chatterjee A.K. (1999). *kdgR_Ecc_* negatively regulates genes for pectinases, cellulase, protease, harpin_Ecc_, and a global RNA regulator in *Erwinia carotovora* subsp. c*arotovora*. J. Bacteriol..

[29] Kazemi-Pour N., Condemine G., Hugouvieux-Cotte-Pattat N. (2004). The secretome of the plant pathogenic bacterium *Erwinia chrysanthemi*. Proteomics.

[30] Tarasova N., Gorshkov V., Petrova O., Gogolev Y. (2013). Potato signal molecules that activate pectate lyase synthesis in *Pectobacterium atrosepticum* SCRI1043. World J. Microbiol. Biotechnol..

[31] Petrova O., Parfirova O., Gogoleva N., Vorob’ev V., Gogolev Y., Gorshkov V. (2023). The role of intercellular signaling in the regulation of bacterial adaptive proliferation. IJMS.

[32] Gorshkov V., Parfirova O., Petrova O., Gogoleva N., Kovtunov E., Vorob’ev V., Gogolev Y. (2021). The knockout of enterobactin-related gene in *Pectobacterium atrosepticum* results in reduced stress resistance and virulence towards the primed plants. IJMS.

[33] Petrova O., Semenova E., Parfirova O., Tsers I., Gogoleva N., Gogolev Y., Nikolaichik Y., Gorshkov V. (2023). RpoS-regulated genes and phenotypes in the phytopathogenic bacterium *Pectobacterium atrosepticum*. IJMS.

[34] Sokolova D.O., Królicka A., Czajkowski R. (2024). Elicitation of potato plants to increase their resistance against Soft Rot *Pectobacteriaceae* bacteria. Eur. J. Plant Pathol..

[35] Rygielska-Tokarska D., Andrei G., Schols D., Snoeck R., Głowacka I.E. (2016). Synthesis, antiviral, cytotoxic and cytostatic evaluation of N 1-(Phosphonoalkyl)Uracil derivatives. Monatsh. Chem..

[36] Głowacka I.E., Piotrowska D.G., Andrei G., Schols D., Snoeck R., Wróblewski A.E. (2016). Acyclic nucleoside phosphonates containing the amide bond. Monatsh. Chem..

[37] Ferreira R., Pires P., De Castro B., Sá Ferreira R.A., Carlos L.D., Pischel U. (2004). Zirconium organophosphonates as photoactive and hydrophobic host materials for sensitized luminescence of Eu(_iii_), Tb(_iii_), Sm(_iii_) and Dy(_iii_). New J. Chem..

[38] Sevrain C.M., Berchel M., Couthon H., Jaffrès P.-A. (2017). Phosphonic acid: Preparation and applications. Beilstein J. Org. Chem..

[39] Ntai I., Manier M.L., Hachey D.L., Bachmann B.O. (2005). Biosynthetic origins of C-P bond containing tripeptide K-26. Org. Lett..

[40] Yamato M., Koguchi T., Okachi R., Yamada K., Nakayama K., Kase H., Karasawa A., Shuto K. (1986). K-26, a novel inhibitor of angiotensin I converting enzyme produced by an *Actinomycete* K-26. J. Antibiot..

[41] Hirayama N., Kasai M., Shirahata K. (1991). Structure and conformation of a novel inhibitor of angiotensin I converting enzyme—A tripeptide containing phosphonic acid. Int. J. Pept. Protein Res..

[42] Kido Y., Hamakado T., Anno M., Miyagawa E., Motoki Y., Wakamiya T., Shiba T. (1984). Isolation and characterization of I5B2, a new phosphorus containing inhibitor of angiotensin I converting enzyme produced by *Actinomadura* sp. J. Antibiot..

[43] Gao J., Ju K., Yu X., Velásquez J.E., Mukherjee S., Lee J., Zhao C., Evans B.S., Doroghazi J.R., Metcalf W.W. (2014). Use of a phosphonate methyltransferase in the identification of the fosfazinomycin biosynthetic gene cluster. Angew. Chem. Int. Ed..

[44] Wilson J., Cui J., Nakao T., Kwok H., Zhang Y., Kayrouz C.M., Pham T.M., Roodhouse H., Ju K.-S. (2023). Discovery of antimicrobial phosphonopeptide natural products from *Bacillus velezensis* by genome mining. Appl. Environ. Microbiol..

[45] Parkinson E.I., Erb A., Eliot A.C., Ju K.-S., Metcalf W.W. (2019). Fosmidomycin biosynthesis diverges from related phosphonate natural products. Nat. Chem. Biol..

[46] Takahashi E., Kimura T., Nakamura K., Arahira M., Iida M. (1995). Phosphonothrixin, a novel herbicidal antibiotic produced by *Saccharothrix* sp. ST-888. I. taxonomy, fermentation, isolation and biological properties. J. Antibiot..

[47] Borisova M., Gisin J., Mayer C. (2014). Blocking peptidoglycan recycling in *Pseudomonas aeruginosa* attenuates intrinsic resistance to fosfomycin. Microb. Drug Resist..

[48] Wild A., Ziegler C. (1989). The effect of bialaphos on ammonium-assimilation and photosynthesis I. effect on the enzymes of ammonium-assimilation. Z. Naturforsch. C.

[49] Mäe A., Montesano M., Koiv V., Palva E.T. (2001). Transgenic plants producing the bacterial pheromone N-acyl-homoserine lactone exhibit enhanced resistance to the bacterial phytopathogen *Erwinia carotovora*. Mol. Plant Microbe Interact..

[50] Chatterjee S., Wistrom C., Lindow S.E. (2008). A cell–cell signaling sensor is required for virulence and insect transmission of *Xylella fastidiosa*. Proc. Natl. Acad. Sci. USA.

[51] Ionescu M., Baccari C., Da Silva A.M., Garcia A., Yokota K., Lindow S.E. (2013). Diffusible signal factor (DSF) synthase RpfF of *Xylella fastidiosa* is a multifunction protein also required for response to DSF. J. Bacteriol..

[52] Hense B.A., Kuttler C., Müller J., Rothballer M., Hartmann A., Kreft J.-U. (2007). Does efficiency sensing unify diffusion and quorum sensing?. Nat. Rev. Microbiol..

[53] Hofer U. (2016). Biofilms: Turning tides for quorum sensing. Nat. Rev. Microbiol..

[54] Li Y.-H., Tian X. (2012). Quorum sensing and bacterial social interactions in biofilms. Sensors.

[55] Huynh T.V., Dahlbeck D., Staskawicz B.J. (1989). Bacterial blight of soybean: Regulation of a pathogen gene determining host cultivar specificity. Science.

[56] Metcalf W.W., Ju K.-S., Jiangtao G.A.O., Doroghazi J.R., van der Donk W.A. (2018). Phosphonic Acid Compounds and Screening Method. U.S. Patent.

[57] Kaniga K., Delor I., Cornelis G.R. (1991). A wide-host-range suicide vector for improving reverse genetics in Gram-negative bacteria: Inactivation of the *blaA* gene of *Yersinia enterocolitica*. Gene.

[58] Quan J., Tian J. (2009). Circular polymerase extension cloning of complex gene libraries and pathways. PLoS ONE.

[59] Evans B.S., Zhao C., Gao J., Evans C.M., Ju K.-S., Doroghazi J.R., Van Der Donk W.A., Kelleher N.L., Metcalf W.W. (2013). Discovery of the antibiotic phosacetamycin via a new mass spectrometry-based method for phosphonic acid detection. ACS Chem. Biol..

[60] Bellincampi D., Dipierro N., Salvi G., Cervone F., De Lorenzo G. (2000). Extracellular H_2_O_2_ induced by oligogalacturonides is not involved in the inhibition of the auxin-regulated *rolB* gene expression in tobacco leaf explants. Plant Physiol..

[61] Shevchik V.E., Robert-Baudouy J., Hugouvieux-Cotte-Pattat N. (1997). Pectate lyase PelI of *Erwinia chrysanthemi* 3937 belongs to a new family. J. Bacteriol..

[62] Miller G.L. (1959). Use of dinitrosalicylic acid reagent for determination of reducing sugar. Anal. Chem..

[63] Seneesrisakul K., Guralp S.A., Gulari E., Chavadej S. (2017). *Escherichia coli* expressing endoglucanase gene from Thai higher termite bacteria for enzymatic and microbial hydrolysis of cellulosic materials. Electron. J. Biotechnol..

[64] Kühnel S., Schols H.A., Gruppen H. (2011). Aiming for the complete utilization of sugar-beet pulp: Examination of the effects of mild acid and hydrothermal pretreatment followed by enzymatic digestion. Biotechnol. Biofuels.

[65] Wrolstad R.E., Wrolstad R.E. (2000). Current Protocols in Food Analytical Chemistry.

[66] Bell K.S., Sebaihia M., Pritchard L., Holden M.T.G., Hyman L.J., Holeva M.C., Thomson N.R., Bentley S.D., Churcher L.J.C., Mungall K. (2004). Genome sequence of the enterobacterial phytopathogen *Erwinia carotovora* subsp. a*troseptica* and characterization of virulence factors. Proc. Natl. Acad. Sci. USA.

[67] Herrero M., De Lorenzo V., Timmis K.N. (1990). Transposon vectors containing non-antibiotic resistance selection markers for cloning and stable chromosomal insertion of foreign genes in Gram-negative bacteria. J. Bacteriol..

[68] Grinter N.J. (1983). A broad-host-range cloning vector transposable to various replicons. Gene.

[69] Datsenko K.A., Wanner B.L. (2000). One-step inactivation of chromosomal genes in *Escherichia coli* K-12 using PCR products. Proc. Natl. Acad. Sci. USA.

