# Supplementary material for: Phosphonates of Pectobacterium atrosepticum: Discovery and Role in Plant–Pathogen Interactions
Source: Int J Mol Sci. 2024 Oct 26;25(21):11516. doi: 10.3390/ijms252111516 (PMC11546328; doi:10.3390/ijms252111516)
Supplement: Supplementary file 1 [file ijms-25-11516-s001.zip › ijms-3261969-supplementary.pdf]

Table S1. Bacterial strains, plasmids and primers used in this study.

**Name**	**Description**	**Origin**
	**Strain**	
Pectobacterium atrosepticum SCRI1043 (*Pba*)	Wild type	[66]
Pectobacterium atrosepticum SCRI1043 Δ*fom1*	Mutant strain of SCRI1043 containing the kanamycin cassette in the chromosome; *Km*^{R}	This study
Pectobacterium atrosepticum SCRI1043 Δ*fom1* complementation mutant	Mutant strain of SCRI1043 containing the kanamycin cassette in the chromosome and the *fom1* complementation construct on pGEM:*fom1*; *Km*^{R} *Amp*^{R}	This study
Pectobacterium atrosepticum SCRI1043 Δ*expI*	Mutant strain of SCRI1043 containing the kanamycin cassette in the chromosome; *Km*^{R}	[31]
Escherichia coli cc118	Host for suicidal vector pKNG101; *Δ(ara, leu) araD ΔlacX 74 galE galK PhoA20 thi-1 rpsE rpoB argE (am) recA1, Sm*^{R}	[67]
Escherichia coli HH26/pNJ5000	Mobilizing strain for conjugative transfer of the suicide vector pKNG101 into *Pba* cells; *tra+; Tet*^{R}	[68]
Escherichia coli NovaBlue	*endA 1hsdR17 (rK12–mK12+) supE44 thi-1 recA1 gyrA96 relA1 lacF' [proA+ B+ lacIqZ ΔM15::Tn10 (Tet*^{R}*)]*	Novagen
	**Plasmids**	
pGEM-T Easy	Linearized vector for cloning; *f1 ori Amp*^{R} *lacZ*	Promega
pGEM:*fom1*	*f1 ori Amp*^{R} *lacZ fom1*	This study
pGEM:Δ*fom1*;Km^{R}	*f1 ori Amp*^{R} *lacZ Km*^{R}	This study
pKD4	Matrix for PCR amplification of kanamycin resistance cassette; *oriRγ rgnB bla Km*^{R}	[69]
pKNG101	Suicide mobilized vector for inactivation of target genes; *pir-ori R6K mobRK2 sacB Sm*^{R}	[57]
pKNG101:Δ*fom1*;Km^{R}	Suicide plasmid carrying mutant locus Δ*fom1*;Km^{R}; *Km*^{R} *SmR sacB*	This study
pGEM:*fom1;* complementation construct	*f1 ori Amp*^{R} *lacZ fom1*	This study
	**Primers**	
**Primer name**	**Primer sequence 5'–3'**	
	**Primers for mutagenesis**	
**up***fom1*F	CATCCAGCGGTCGTCTTCTACA	
**dn***fom1*R	CATCCCCATCCAGACAGACAAC	
**dn***fom1*KmF	CCATGTCAGCCGTTAAGCCGGAACGCGCTAAC	
**up***fom1*KmR	CAGCTCCAGCCTACACAATCGTAGGTAAAATGATAGACATG	
**Km***fom1*F	CTATCATTTTACCTACGATTGTGTAGGCTGGAGCTGCTTC	
**Km***fom1*R	GCGCGTTCCGGCTTAACGGCTGACATGGGAATTAGC	
**Check***fom1*F	GGCGTGAAAAAGTCAGGGCAGG	
**Check***fom1*R	GGTGCCAGCGGTAAGTGGGAC	
**T7F**	TAATACGACTCACTATAGGG	

**Sp6R**	TAAATCCACTGTGATATCTTA
**Primers for complementation construct**	
**comp***fom1*F	GCCCTCTCTTTATCTCCCATCC
**comp***fom1*R	CCTTCGCATCGTGCTGTATTTAACAG
**Primers for qRT-PCR**	
*fom1*_F	CAGCCATCGTTTCCATGCG
*fom1*_R	CAGCAGCCCGAATGTGGTG
*rpoD*_F	CTAATGCGGAAGAAGACATTGCCCC
*rpoD*_R	CTTCTCACGCGCCAGTTCAGGATCG
*Ffh*_F	GTGGCATCATCCCTTTCATACCGC
*Ffh*_R	CGATTCAGGAACGTGCTAAACCAGAG
*recA*_F	GTGGATATTGATGCCTTCGCCG
*recA*_R	GTTTTATGCCTCTGTTCGTTTGGATATTC
